# An easy and efficient inducible CRISPR/Cas9 platform with improved specificity for multiple gene targeting

**DOI:** 10.1093/nar/gkw660

**Published:** 2016-07-25

**Authors:** Jian Cao, Lizhen Wu, Shang-Min Zhang, Min Lu, William K.C. Cheung, Wesley Cai, Molly Gale, Qi Xu, Qin Yan

**Affiliations:** 1 Department of Pathology, Yale School of Medicine, New Haven, CT, 06520 USA; 2 Harbin Institute of Technology, Harbin, 150001 China; 3 Department of Oncology, Renji Hospital, Shanghai Jiao Tong University School of Medicine, Shanghai, 200127 China

## Abstract

The CRISPR/Cas9 system is a powerful genome editing tool and has been widely used for biomedical research. However, many challenges, such as off-target effects and lack of easy solutions for multiplex targeting, are still limiting its applications. To overcome these challenges, we first developed a highly efficient doxycycline-inducible Cas9-EGFP vector. This vector allowed us to track the cells for uniform temporal control and efficient gene disruption, even in a polyclonal setting. Furthermore, the inducible CRISPR/Cas9 system dramatically decreased off-target effects with a pulse exposure of the genome to the Cas9/sgRNA complex. To target multiple genes simultaneously, we established simple one-step cloning approaches for expression of multiple sgRNAs with improved vectors. By combining our inducible and multiplex genome editing approaches, we were able to simultaneously delete Lysine Demethylase (KDM) 5A, 5B and 5C efficiently *in vitro* and *in vivo*. This user friendly and highly efficient toolbox provides a solution for easy genome editing with tight temporal control, minimal off-target effects and multiplex targeting.

## INTRODUCTION

The discovery and engineering of the CRISPR/Cas9 system in the past several years has revolutionized genome editing techniques and functional genetics studies (1). Programming of the Cas9 endonuclease with an RNA chimera (single guide RNA or sgRNA), which comprises a direct sequence of 20 nucleotides (nt) and an RNA scaffold, is sufficient to generate double-strand breaks in a targeted DNA locus (2–4). In mammalian cells, double-strand breaks are repaired through a pathway called non-homologous end-joining, which often leads to small insertions or deletions (indels) (2,3). Thus, the CRISPR/Cas9 system provides a simple way to abolish expression of selected proteins by shifting the reading frame or disrupting splicing sites.

Inducible strategies for CRISPR/Cas9-based genome editing have been developed recently. Some of these studies used knock-in techniques to introduce doxycycline (DOX)-inducible Cas9 (iCas9) into certain DNA loci (5–8). Knock-in is based on homologous recombination, so that cloning of a specific DNA sequence is required. Other studies used various inducible methodologies, such as Tet-off controlled sgRNA (9), heat-shock iCas9 (10), photoactivatable or chemiactivatable Cas9 (11–13) and split Cas9 (14,15). Wang *et al*. used a DOX-iCas9 vector and selected monoclonal cells to perform inducible gene silencing (16). However, there is no publicly available construct to introduce iCas9 into a large panel of cell lines with high efficiency in a polyclonal setting, which can significantly decrease time needed for selection of single clones for phenotypic analysis and avoid clonal variations.

The CRISPR/Cas9 system can be engineered to disrupt multiple genes simultaneously by co-expressing multiple sgRNAs carrying different direct sequences. It can be done by applying a CRISPR RNA array and tracrRNA separately (2), injecting or transfecting multiple *in vitro* prepared sgRNA molecules (5,17), using multiple single sgRNA plasmids (18,19) or using a single plasmid to deliver multiple sgRNAs targeting different coding genes (7,20–23). The last option has obvious experimental advantages in most cases. However, complicated cloning steps or multiple sgRNA expression vectors are often required (5–7,9,18,21,24,25). Vidigal *et al*. developed a method to clone a pair of sgRNAs into one plasmid (20). Dow *et al*. used a multi-step strategy to introduce sgRNA cassettes sequentially (7). Kabadi *et al*. developed a method to generate a plasmid with four sgRNA cassettes, which requires cloning sgRNAs into four specific vectors beforehand (21). Therefore, a simpler strategy for multiple gene targeting will broaden the usage of CRISPR/Cas9-based genome editing.

Off-targeting is one of the major challenges for gene silencing approaches. The specificity of genome editing introduced by CRISPR/Cas9 is based on a 20-nt guide sequence. It directs Cas9 to the genomic target by Watson–Crick base pairing. However, recent studies (26–29) have demonstrated that mismatches between guide RNAs and their targets can be tolerated, leading to genome editing at unexpected sites. Different approaches have been developed to enhance the specificity of CRISPR/Cas9 mediated genome editing. These methods include avoiding guide sequences with known off-target sites (29,30), shortening guide sequences (31), pairing double nicking (32) and engineering Cas9 (33–35). Here we report a new strategy to decrease off-target effects by limiting Cas9 exposure with DOX-iCas9, which also allows for uniform temporal control of genome editing.

Given the advantages provided by an inducible CRISPR/Cas9 system and multiplex targeting, there is an urgent need for a uniform, highly efficient and easy solution to combine these two approaches. To address this need, we developed a highly efficient CRISPR/Cas9 platform for inducible and multiplex genome editing. As a proof of concept, we have successfully generated inducible KDM5A, KDM5B and KDM5C triple knockout cells using our platform. Furthermore, this system allowed us to significantly shorten induction time, resulting in dramatically decreased off-target effects. Therefore, this system is an attractive solution that allows for easy and clean genome editing.

## MATERIALS AND METHODS

### Plasmid constructs

Human codon-optimized *Streptococcus pyogenes* Cas9 (spCas9) and *porcine teschovirus-1* 2A peptide (P2A) self-cleavage sequence (36) were polymerase chain reaction (PCR) amplified from LentiCRISPRv1 plasmid (37) and cloned into pcDNA3 (Invitrogen) upstream of the EGFP coding sequence. Cas9-P2A-EGFP was then cloned into pINDUCER-20 (38) by Gateway recombination to generate Lenti-iCas9-neo. Lac-Cm^r^-ccdB cassette was PCR amplified from pINDUCER-20. U6 promoter and sgRNA scaffold were PCR amplified from LentiGuide (39). LentiCRISPRv2 and LentiGuide (39) were digested by BsmBI. LentiGuide without sgRNA cassette was PCR amplified. Golden Gate Assembly was then performed with BsmBI digested Lac-Cm^r^-ccdB, sgRNA scaffold, U6 promoter and LentiCRISPRv2 to generate Lenti-multi-CRISPR, with BsmBI digested Lac-Cm^r^-ccdB, sgRNA scaffold, U6 promoter and LentiGuide to generate Lenti-multi-Guide and with BsmBI digested Lac-Cm^r^-ccdB and LentiGuide backbone to generate Lenti-entry-puro. sgRNAs were designed using Feng Zhang lab's server (http://crispr.mit.edu/) or CHOPCHOP (https://chopchop.rc.fas.harvard.edu/) (40), and cloned into LentiGuide or LentiCRISPRv1 (37) to generate single sgRNA carrying plasmids. For generation of multiple sgRNA carrying plasmids, the fragments of sgRNA scaffold and U6 promoter were amplified from Lenti-multi-CRISPR or Lenti-multi-Guide. Guide sequences and BsmBI sites were added into these primers as listed in Supplementary Table S2. For generation of multiple sgRNA carrying plasmids based on sgRNA delivery plasmids, sgRNA cassettes were PCR amplified with the primers listed in Supplementary Table S3. In both cases, the PCR reactions were performed with Phusion polymerase (NEB) with the following program: preheat at 98°C for 60 s, 35 cycles of 3-step amplification (98°C for 15 s, 52°C for 15 s, 72°C for 30 s), and final extension at 72°C for 60 s. PCR products and vectors (Lenti-multi-CRISPR, Lenti-multi-Guide or Lenti-entry-puro) were digested by BsmBI (NEB) and purified from agarose gel after separation by electrophoresis. Ligation reactions were performed with equal molar amounts of vector and insert fragments. T4 DNA ligase (NEB) was used following the manufacturer's protocol. Ligation products were transformed into 50 μl Stbl3 competent cells (10^7^ cfu/μg) by heat shock and 1/10 of the bacteria was spread on LB plates with 100 μg/ml carbenicillin. Bacterial clones were counted to calculate the ligation efficiency. To assess the ratio of correctly assembled constructs, 10 randomly picked clones per reaction were selected for plasmids purification. The size of insertion in these plasmids was verified by restriction enzyme digestion with NotI and XhoI. Eight restriction digestion verified plasmids, including three of six sgRNA plasmids and five of three sgRNA plasmids, were further verified by Sanger sequencing. LentiGuide (39) was used to deliver single sgRNAs. Lenti-entry-puro was used to deliver sgRNAs targeting KDM5A, 5B and 5C. LentiCRISPRv1 (37) was used to deliver constitutively expressed Cas9 and single sgRNAs. All vectors will be deposited into the Addgene repository.

### Cell culture

293T, HeLa and SKBR3 cells were maintained in Dulbecco's modified Eagle's medium (DMEM) supplemented with 10% fetal bovine serum (FBS). BT474, MCF7, PC9 and NT2 cells were maintained in RPMI1640 supplemented with 10% FBS. MCF10A cells were maintained in DMEM/F12 supplemented with 5% horse serum, 20 ng/ml EGF, 0.5 mg/ml hydrocortisone, 100 ng/ml cholera toxin and 10 μg/ml insulin. For lentivirus production, 293T cells in 12-well plates were transfected with Lipofectamine 2000 (Invitrogen), according to the manufacturer's protocol. For each well, 750 ng lentiviral plasmid, 500 ng psPAX2 and 250 ng pMD2.G were used. Twenty-four hours after transfection, 293T cells were refed with fresh medium. Forty-eight hours after transfection, lentivirus-containing medium was collected and filtered through a 0.45 μm filter before being used to infect cells. For generation of stable cell lines, cells were infected with lentivirus for 24 h, then refed with fresh medium with the selection drug. Cells were refed every 2 days until uninfected control cells were completely killed. Killing took 4 days for puromycin and 7 days for G418. To sort for cells with high Cas9 induction efficiency, SKBR3/iCas9, HeLa/iCas9, and MCF10A/iCas9 cells were treated with 1 μg/ml DOX for 24 h and EGFP positive cells were sorted out by FACS. To generate single clones of HeLa/iCas9 cells, single cells were seeded into 96-well plates. Cells grown up from a single cell were picked up and EGFP induction was tested again. One cell line (HeLa/iCas9-c1) with the best EGFP induction was selected and used in this manuscript.

### Immunoblot analyses

Protein extraction and immunoblotting were performed as described previously (41,42). KDM5A antibody (D28B10) was purchased from Cell Signaling, KDM5B antibody (HPA027179) was purchased from Sigma and KDM5C antibody (A301-035A) was purchased from Bethyl Laboratories. Tubulin (T5168) and vinculin (V9131) antibodies were purchased from Sigma. H3K4me3 (ab8580) and H3 (ab1791) antibodies were purchased from Abcam. ARID2 antibody (sc-166117) was purchased from Santa Cruz Biotechnology. Quantification was performed with ImageJ (https://imagej.nih.gov/). All results were normalized to loading controls (vinculin or tubulin) and the ratios to non-induced control (for iCas9) or control sgRNA (constitutively expressed Cas9) were calculated and presented as a percentage of the controls.

### T7 endonuclease assays

Genomic DNA was extracted using the DNeasy blood and tissue kit (Qiagen) following the manufacturer's protocol. The genomic region surrounding the target sites or predicted off-target sites for each guide sequence was PCR amplified with Phusion polymerase (NEB) with the following program: preheat at 98°C for 60 s, 35 cycles of 3-step amplification (98°C for 15 s, 62°C for 15 s, 72°C for 30 s) and final extension at 72°C for 60 s. The primers used are listed in Supplementary Table S5. A total of 200 ng of the purified PCR products were mixed with Buffer 2 (NEB) and ultrapure water to a final volume of 19 μl. Hybridization reactions were performed with the following program: 95°C for 5 min; ramp down to 85°C at −2°C/s; ramp down to 25°C at −0.1°C/s. Then 1 μl T7 endonuclease I (NEB) was added and the mixture was incubated at 37°C for 1 h. A total of 2 μl of 0.25M ethylenediaminetetraacetic acid was added to stop the reaction followed with gel electrophoresis on a 2% agarose gel. Quantification was performed with ImageJ (https://imagej.nih.gov/). The percentage of indels was calculated as described previously (43).

### Animal experiments

Studies were conducted in compliance with US guidelines for the care and use of laboratory animals and were approved by the Institutional Animal Care and Use Committee of Yale University. 5 × 10^5^ HeLa/iCas9-c1 cells carrying control sgRNA or KDM5A/B/C sgRNAs were subcutaneously injected into the flanks of the 6-week-old female nude mice (Athymic NCR-nu/nu, Charles River Laboratories). After the tumors reached the size of ∼0.5 cm^3^, the animals were fed with normal chow or chow containing 625 mg/kg DOX for 5 days before euthanizing and harvesting tumor tissues for western blot analyses.

## RESULTS

### A robust Tet-on Cas9 expression vector with EGFP reporter

**Figure 1.**
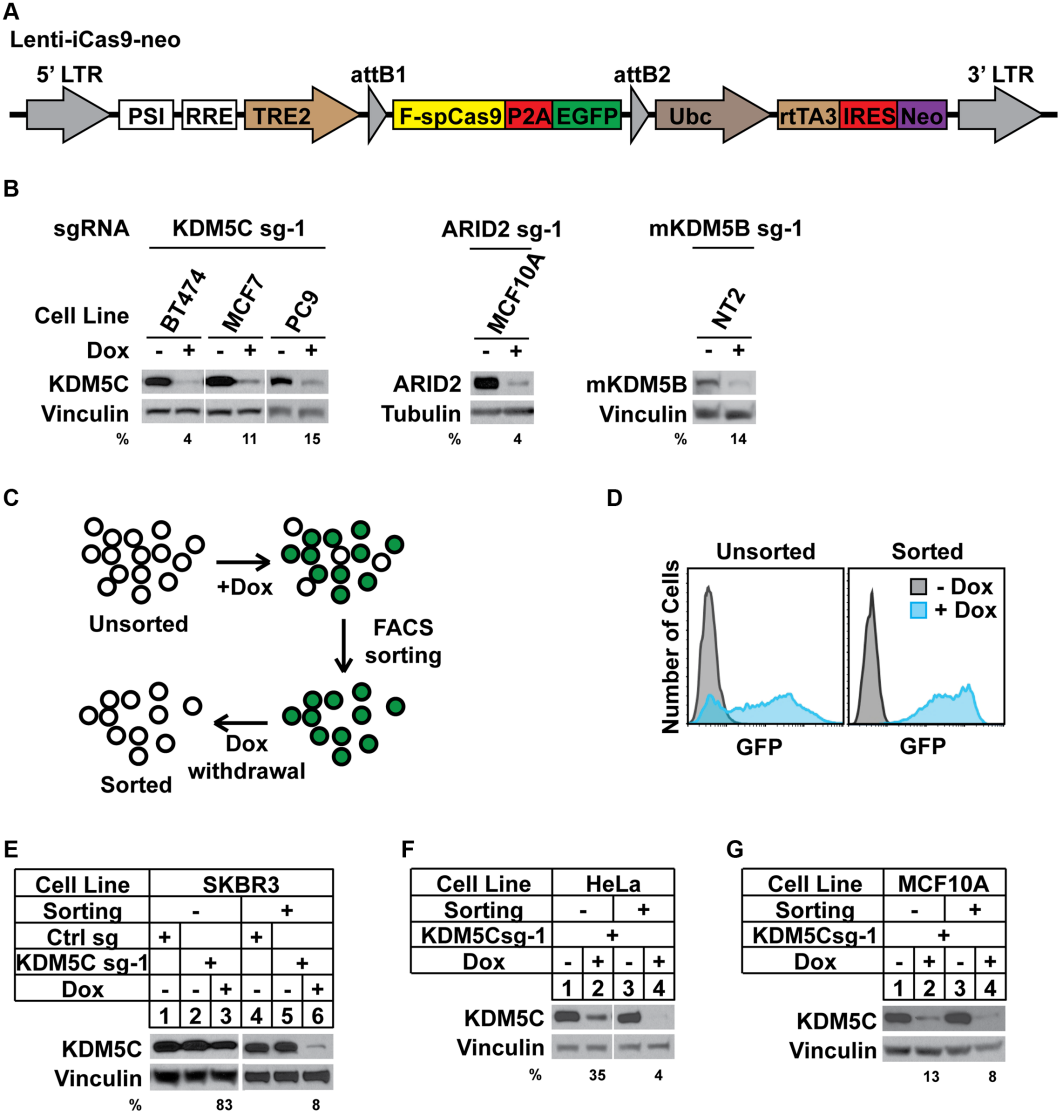
A sensitive and efficient inducible CRISPR/Cas9 system. (**A**) Schematic representation of the Lenti-iCas9-neo plasmid. LTR, long terminal repeat; PSI, retroviral Ψ packaging element; RRE, Rev response element; TRE2, TRE2 promoter; P2A, a 2A self-cleavage peptide from *porcine teschovirus-1*; Ubc, Ubiquitin C promoter; rtTA3, reverse tetracycline-controlled transactivator 3; IRES, internal ribosome entry site; Neo, neomycin resistance. (**B**) The indicated cells were transduced with Lenti-iCas9-neo and LentiGuide carrying the indicated sgRNA. Cells were then treated with or without 1 μg/ml doxycycline (DOX) for 72 h and analyzed by western blots. (**C**) Overview of the FACS sorting strategy. (**D**) Unsorted and sorted HeLa/iCas9 cells were treated with or without 1 μg/ml DOX for 24 h and GFP expression was analyzed by FACS. (**E–G**) SKBR3 (E), Hela (F) and MCF10A (G) iCas9 cells before or after FACS sorting were transduced with LentiGuide carrying control or KDM5C sgRNA. Cells were then treated with or without 1 μg/ml DOX for 72 h and analyzed by western blots.

To develop an iCas9 system, we took advantage of the highly efficient DOX-inducible pINDUCER system, which has been shown to be broadly inducible in various cell types *in vitro* and *in vivo* (38). We selected the pINDUCER-20 lentiviral vector, which allows for neomycin selection. To facilitate monitoring of Cas9 expression, we introduced an EGFP reporter downstream of FLAG-tagged spCas9, separated by a P2A self-cleavage sequence (36) (Figure 1A). To test the efficiency of Cas9 mediated genome editing, multiple iCas9 stable cell lines were generated, including human breast cancer cell lines BT474, MCF7 and SKBR3, human immortalized mammary epithelial cell line MCF10A, human non-small cell lung cancer cell line PC9 and mouse breast cancer cell line NT2 (44) (Supplementary Table S1). sgRNAs targeting human KDM5C, ARID2 or mouse KDM5B were then introduced into these iCas9 cell lines. The expression of targeted proteins was decreased to 4–15% after DOX treatment in these cell lines (Figure 1B).

To further enhance the gene silencing efficiency, we developed a strategy to enrich for cells with tighter DOX-controlled expression of Cas9. Briefly, iCas9 cells were treated with DOX followed by FACS sorting for EGFP positive cells (Figure 1C). As demonstrated in the human cervical cancer cell line HeLa, the sorted iCas9 cells were 100% EGFP positive after DOX induction (Figure 1D). We then compared genome editing efficiency between unsorted and sorted polyclonal populations using three cell lines: SKBR3, HeLa and MCF10A (Figure 1E–G). Although unsorted SKBR3/iCas9 cells only showed limited decrease of KDM5C expression after treatment with DOX and an sgRNA targeting KDM5C, significant inducible gene silencing was achieved in the sorted SKBR3/iCas9 cells (Figure 1E). Sorted HeLa/iCas9 and MCF10A/iCas9 cells also showed improved inducible genome editing, as shown by the degree of decreased KDM5C expression (Figure 1F and G). Using this strategy, we have also generated sorted iCas9 containing MCF7 and BT474 cell lines (Supplementary Table S1). These results indicated that one simple sorting step can further improve genome editing in the polyclonal setting.

### One-step cloning strategies to generate multi-sgRNA carrying plasmids

**Figure 2.**
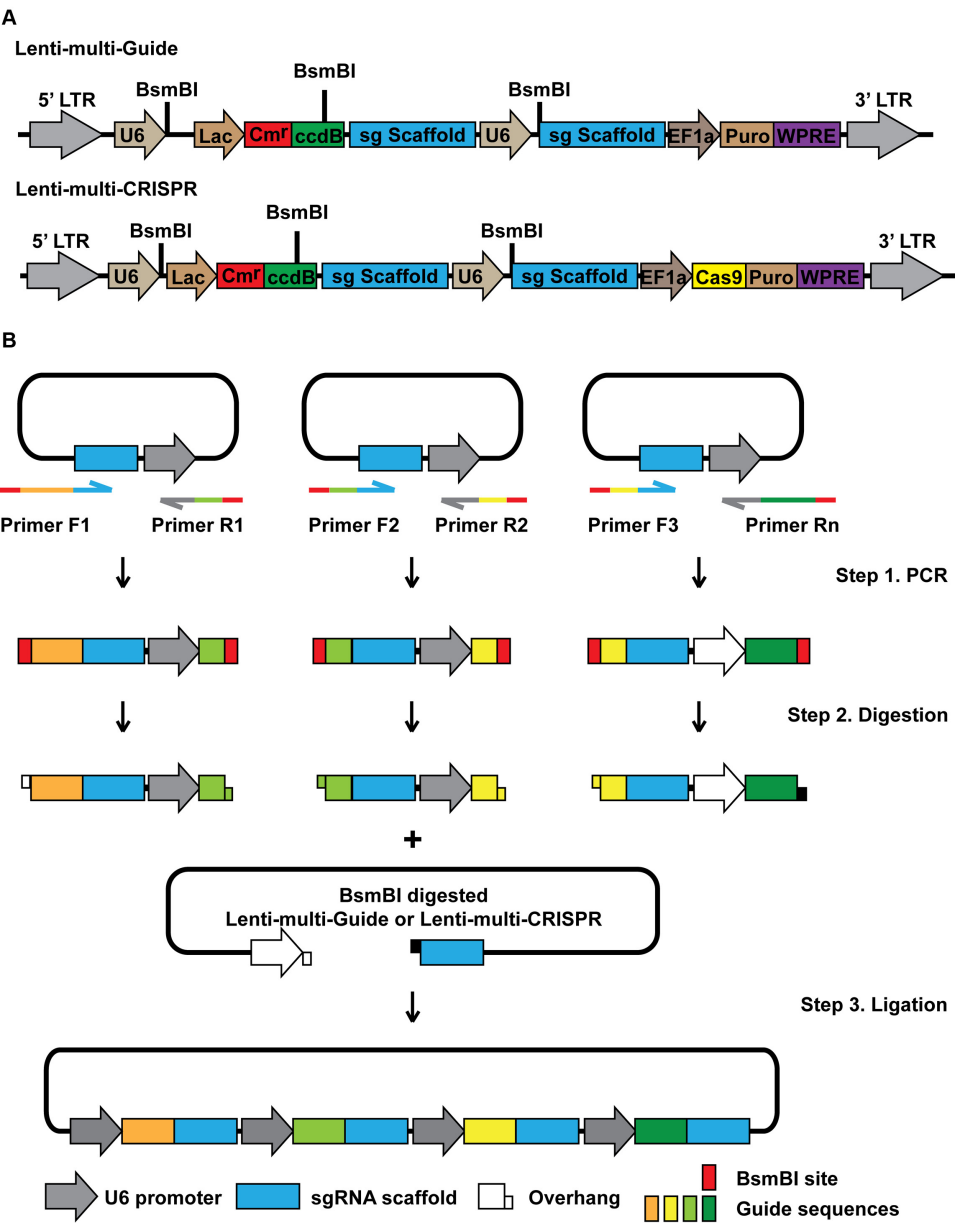
A one-step method to generate a multiple sgRNA delivery plasmid. (**A**) Schematic representation of the Lenti-multi-Guide and Lenti-multi-CRISPR plasmids. LTR, long terminal repeat; U6, U6 promoter; Lac, Lac promoter; Cm^r^, Chloramphenicol resistance; ccdB, toxin ccdB gene; sg Scaffold, sgRNA scaffold sequence; EF1a, EF1α promoter; Cas9, spCas9; Puro, puromycin resistance; WPRE, Woodchuck hepatitis virus post-transcriptional regulatory element. Retroviral Ψ packaging element and Rev response element were not shown. (**B**) Overview of the cloning strategy.

The CRISPR/Cas9 system has been applied to target multiple genes by introducing several sgRNAs at the same time. Current strategies of generating multiple sgRNA delivery plasmids include multi-step sub-cloning (7) or pre-cloning sgRNAs into different vectors (21). To simplify the cloning method, we generated Lenti-multi-Guide and Lenti-multi-CRISPR (Figure 2A), and designed a new strategy based on Golden Gate Assembly (37) (Figure 2B). Briefly, sgRNA scaffold and U6 promoter were amplified by PCR from Lenti-multi-Guide or Lenti-multi-CRISPR. The first and the last guide sequence were introduced into the first forward primer and the last reverse primer, respectively (Supplementary Table S2). Other guide RNAs were split into a pair of forward and reverse primers (Supplementary Figure S1 and Table S2). BsmBI sites were introduced to these primers to allow reconstitution of the guide RNA sequence. BsmBI sites and an overhang sequence were also introduced to the first forward primer and the last reverse primer to allow ligation into Lenti-multi-Guide or Lenti-multi-CRISPR (Figure 2B; Supplementary Figure S1b and Table S2). The resulting Lenti-multi-Guide based plasmids can be used in iCas9 cells for inducible multi-targeting, while the Lenti-multi-CRISPR based constructs can be used for constitutive multi-targeting.

To take advantage of the validated sgRNA delivery plasmids, we also developed another vector, Lenti-entry-puro (Supplementary Figure S2a) and a similar method to generate multiple sgRNA delivery plasmids based on pre-generated single sgRNA delivery plasmids. Briefly, sgRNA cassettes, including promoter, 20 nt targeting sequence and sgRNA scaffold, were amplified by PCR from one to six individual delivery vectors, such as LentiCRISPR or LentiGuide with the sgRNA cassette. BsmBI sites and a different overhang sequence were introduced to primers to allow sequential ligation of multiple sgRNA cassettes (Supplementary Figure S2b and Table S3).

**Figure 3.**
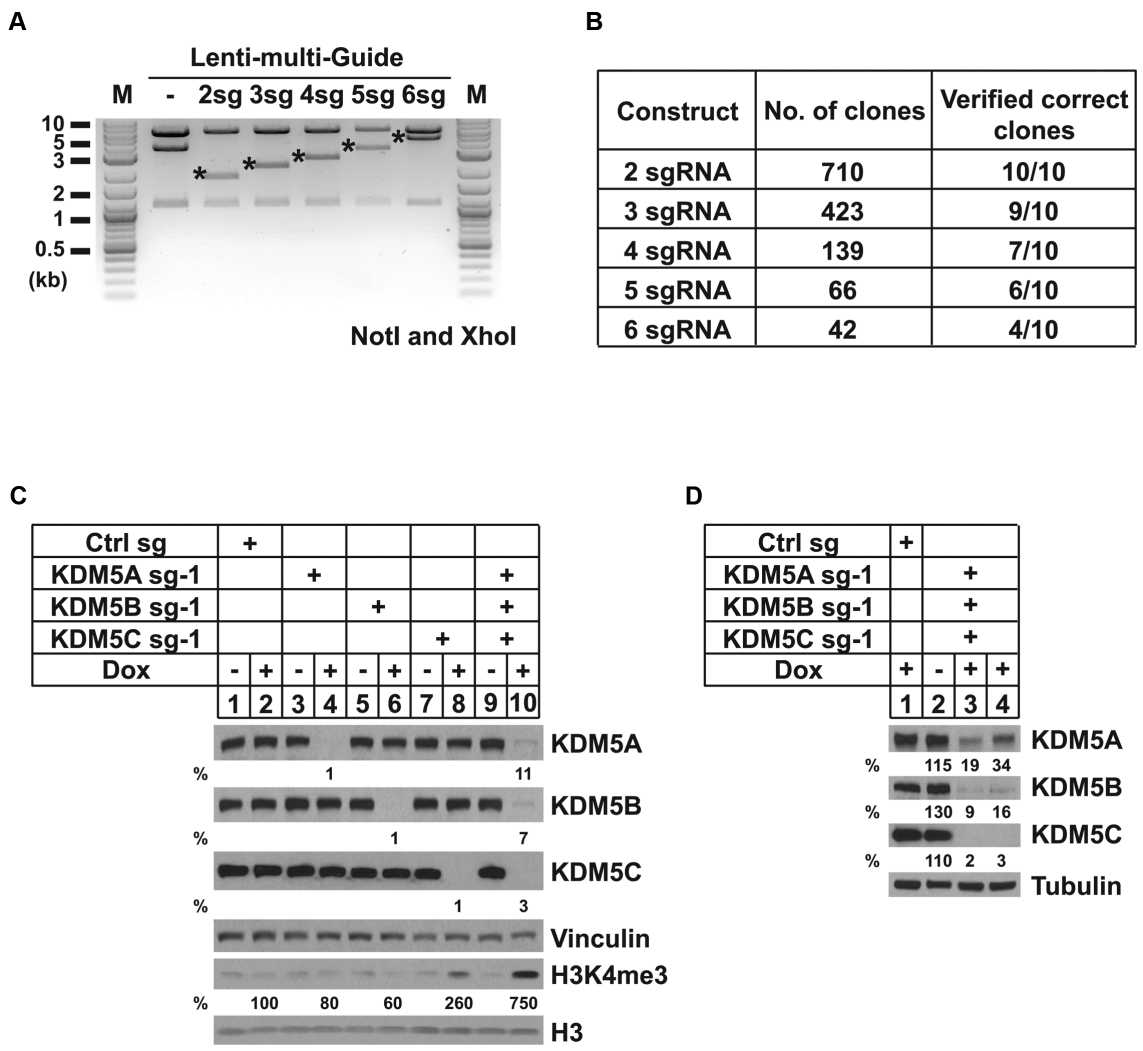
Inducible and multiplexed gene knockout *in vitro* and *in vivo*. (**A**) NotI and XhoI digestion of indicated plasmids showing correct vector assembly of 2, 3, 4, 5 or 6 sgRNA cassettes. LG, LentiGuide. M, Log-2 DNA ladder (NEB). Fragments carrying sgRNA cassettes were indicated by stars (*). (**B**) A summary of the cloning efficiency for making the correct constructs with different numbers of sgRNAs. Ten clones were randomly picked and verified by the size of NotI and XhoI digested fragments. (**C**) HeLa/iCas9-c1 cells were transduced with LentiGuide or Lenti-entry-puro carrying the indicated sgRNA(s). Cells were then treated with or without 1 μg/ml DOX for 48 h and harvested for western blot analyses. (**D**) Western blot analyses of tumor tissues isolated from the mice injected with indicated cells and fed with control or DOX chow.

With both strategies, we were able to obtain constructs for delivery of multiple sgRNAs efficiently. It is worth mentioning that a ccdB suicide gene was introduced into Lenti-entry-puro to decrease background caused by self-ligation or incomplete digestion. CcdB-containing plasmids can only grow in certain strains, such as DB3.1 (45), providing an additional positive selection of cloning. As a proof-of-principle of these cloning strategies, we successfully generated constructs with one to six sgRNA cassettes (Figure 3A and Supplementary Figure S2c). To examine the cloning efficiency, we compared the number of colonies after transformation and the ratio of verified clones by restriction digestion and Sanger sequencing. As expected, the ligation efficiency and the ratio of correct clones negatively correlated with the number of fragments in the ligation reaction (Figure 3B and Supplementary Figure S2d). The mis-ligation may be caused by the toleration of mismatches between the sticky ends of DNA fragments. The success rate was very high even for the construction of a delivery plasmid containing six sgRNAs in one-step cloning (Figure 3B). Furthermore, we selected eight restriction digestion verified plasmids for Sanger sequencing, and all these plasmids were sequencing verified.

### Efficient knockout of KDM5A, 5B and 5C simultaneously *in vitro* and *in vivo*

KDM5 family proteins, KDM5A (RBP2), KDM5B (PLU1), KDM5C (SMCX) and KDM5D (SMCY), are mammalian demethylases for trimethylated lysine 4 of histone H3 (H3K4me3), an epigenetic mark associated with transcriptional activation (46–49). KDM5D is located on the Y chromosome, so only KDM5A, 5B and 5C H3K4me3 demethylases are present in cells isolated from females. To generate H3K4me3 demethylase-depleted cells, we cloned three sgRNAs targeting KDM5A, KDM5B or KDM5C into Lenti-entry-puro and introduced the vector into a monoclonal HeLa/iCas9 cell line (HeLa/iCas9-c1). After DOX induction, single sgRNA carrying cells showed undetectable levels of target proteins, while cells carrying all three sgRNAs showed simultaneous depletion of all three target proteins, indicating the high efficiency of multiplex gene silencing of our system (Figure 3C). Depletion of KDM5A or KDM5B alone did not increase global H3K4me3 levels, while KDM5C depletion increased H3K4me3 level by 160%. In contrast, simultaneous depletion of KDM5A, 5B and 5C increased global H3K4me3 level by 650% (Figure 3C). These results indicated that all three KDM5 enzymes contribute to regulation of global H3K4me3 level in HeLa cells.

After demonstrating the efficiency of this inducible and multiplex genome editing system in cell culture, we then tested our system in a xenograft model. The HeLa/iCas9-c1 cells carrying control or KDM5A/B/C sgRNAs were injected into nude mice subcutaneously. Tumor bearing animals were fed with regular or DOX chow for 5 days, and tumors were analyzed for expression of KDM5s. We found that significant deletion of KDM5A, 5B and 5C were observed in KDM5A/B/C sgRNA containing tumors, compared to the tumors isolated from the mice injected with control cells (Figure 3D, compare lanes 3–4 with lane 1). The deletion efficiency in the tumor cells is likely similar to what was observed in cell culture experiments due to the existence of non-tumor cells in the tumor tissues. Moreover, the tumors isolated from mice fed with regular chow did not show any decrease of the expression of KDM5s, suggesting that Cas9 mediated deletion is tightly controlled (Figure 3D, compare lane 2 with lane 1). These results indicated that our inducible multiplex system worked efficiently *in vitro* and *in vivo*.

### Decreased off-target effects with inducible CRISPR/Cas9 system

**Figure 4.**
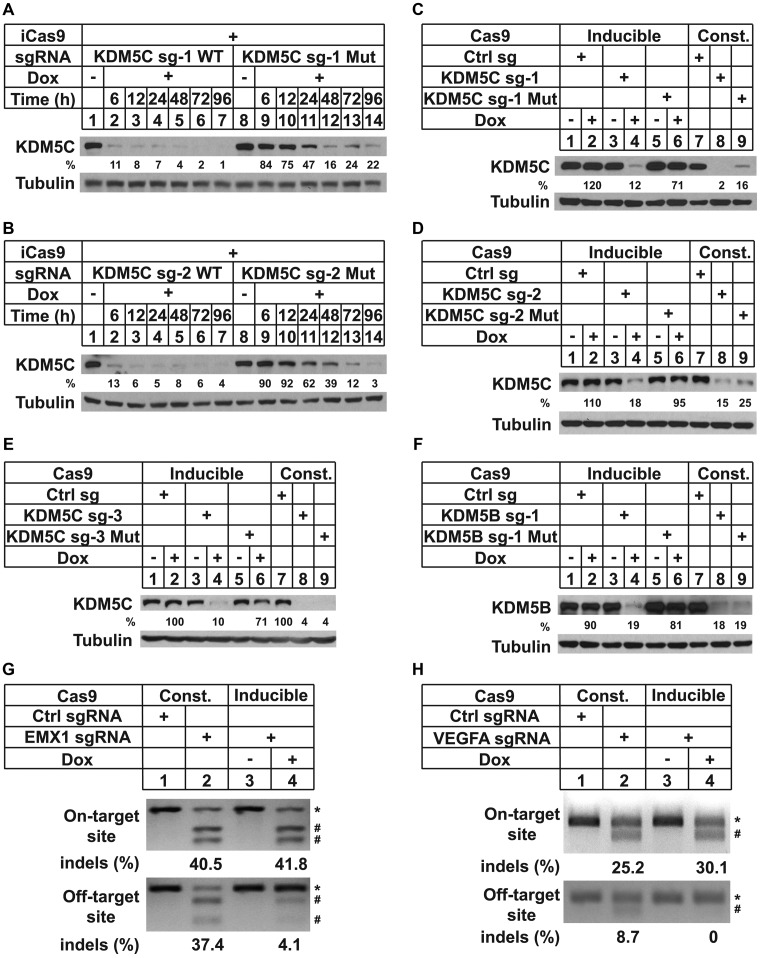
Limiting duration of Cas9 induction reduced potential off-target effects. (**A** and **B**) HeLa/iCas9-c1 cells were transduced with LentiGuide carrying the indicated sgRNA. Cells were then treated with 0.1 μg/ml DOX for the indicated time, harvested after 96 h and analyzed by western blots. (**C–F**) HeLa/iCas9-c1 cells were transduced with LentiGuide carrying the indicated sgRNA, treated with 0.1 μg/ml DOX for 12 h and harvested after 48 h for western blot analyses (for iCas9). HeLa cells were transduced with LentiCRISPRv1 carrying the indicated sgRNA and harvested for western blot analyses within 10 days after transduction (Const., for constitutively expressed Cas9). (**G** and **H**) HeLa/iCas9-c1 cells were transduced with LentiGuide carrying the indicated sgRNA (inducible). HeLa cells were transduced with LentiCRISPRv1 carrying constitutively expressed Cas9 and the indicated sgRNA (Const.). iCas9 cells were then treated with 0.1 μg/ml DOX for 12 h and cultured for another 60 h. All cells were harvested for genomic DNA isolation and analyzed by T7 endonuclease assay. Stars (*) indicate undigested DNA. Pound signs (#) indicate digested products. The second digested fragments in Figure 4H were too short to be visible in same gel.

We hypothesized that shortening the exposure time of the genome to Cas9/sgRNA would decrease off-target effects caused by tolerance of mismatches between sgRNA and genomic loci carrying similar sequences. To test this hypothesis, we introduced mismatches into two independent sgRNAs targeting KDM5C (Supplementary Table S4). Treating cells carrying wild-type (WT) sgRNA with 0.1 μg/ml DOX for 6 h was sufficient for significant decrease of target protein expression in iCas9 cells (Supplementary Figure S3a and b; Figure 4A and B). In contrast, sgRNAs carrying even one single mismatch at the 10th nt (Mut) significantly delayed the decrease of expression (Figure 4A and B). However, after 96 h of induction, cells carrying mutated sgRNA also achieved significant gene silencing (Figure 4A and B). The large difference in the time-dependent genome editing between WT and mutant sgRNAs suggested that limiting Cas9 induction could minimize potential off-target effects while achieving a high efficiency of silencing of the intended target gene. To further test this idea, we compared the gene silencing efficiency of inducible or constitutively expressed Cas9. Wild-type sgRNAs against KDM5C or KDM5B showed similar gene silencing efficiency with both systems. In contrast, mutant sgRNAs only caused significant gene silencing with constitutively expressed Cas9 (Figure 4C–F).

To corroborate our findings, we determined if transient expression of Cas9 lowered undesirable genome editing on some known endogenous off-target sites using T7 endonuclease assays. With constitutively expressed Cas9, the guide sequences targeting EMX1 or VEGFA caused significant off-target effects as reported previously (50) (Figure 4G and H). With iCas9 and limited induction time, the indels at off-target sites were significantly decreased (Figure 4G and H). On the other hand, the efficiencies of genome editing at on-target sites remained comparable to constitutively expressed Cas9 (Figure 4G and H). Collectively, these results indicated that off-target effects are minimized with our inducible system.

## DISCUSSION

Here we provided an easy solution to expand the application of CRISPR/Cas9 mediated gene silencing. This is a user-friendly and highly efficient CRISPR/Cas9 platform generated by combining iCas9 and multiplex sgRNAs. It contains four lentiviral vectors: Lenti-iCas9-neo for delivery of DOX-iCas9 with an EGFP reporter (Figure 1A), Lenti-multi-Guide or Lenti-entry-puro for assembly and delivery of multiple sgRNAs (Figure 2A and Supplementary Figure S2a), and Lenti-multi-CRISPR vector for non-inducible multiplex genome editing (Figure 2A). This genome editing platform allows for tight temporal control, multiplex targeting and decreased off-target effects.

Compared to current inducible strategies for CRISPR/Cas9 introduced gene silencing (5–15), our platform has multiple advantages. Firstly, we achieved nearly complete gene silencing even in the polyclonal setting in most of the cell lines tested (Figure 1B, E and F), which has not been previously reported. Thus, our system will greatly decrease the time needed to generate clonal knockout cells for phenotypic analysis and avoid the concerns of clonal effects. Secondly, the EGFP reporter expressed from the same transcript as Cas9 is a powerful tool to monitor background Cas9 expression and Cas9 induction. The EGFP reporter can also be utilized to sort for cells with extremely high efficiency of Cas9 induction before introducing sgRNAs (Figure 1C). This step can further increase the efficiency of genome editing (Figure 1D–F). In addition, the EGFP reporter simplifies further selection procedures if monoclonal cells are needed. Thirdly, we have shown that our plasmid performed well in both human and mouse cell lines and in a mouse xenograft model (Figure 3D) and is therefore widely applicable.

The two reported multiplex sgRNA assembly systems were designed to satisfy the demands for what current methods are missing. The first strategy starts from guide RNA sequences (Figure 2B). The second strategy is based on pre-generated/validated single sgRNA delivery plasmids (Supplementary Figure S2b). With both strategies, only a single step of cloning is required to generate a multiple-sgRNA delivery plasmid, instead of one step for each sgRNA (7). The number of sgRNAs in the combination is not limited to two (20) or four (21). Furthermore, our second method can be applied to any sgRNA cassette carrying vectors instead of specific vectors used by other methods (21). Additionally, the ccdB suicide gene in the sgRNA assembly vectors (Figure 2A and Supplementary Figure S2a) diminished potential false positive clones to simplify the cloning efforts, as demonstrated by the robust cloning efficiency (Figure 3B and Supplementary Figure S2d). Importantly, our methods can also be used to generate libraries with selected sgRNA combinations for functional screening to identify certain gene–gene interactions such as synthetic lethality.

Off-target effects pose a major challenge for sequence-based approaches, such as RNAi and genome editing. Several strategies to enhance Cas9 specificity have been reported, including searching for guide sequences with fewer potential off-target sites (29,30), using truncated guide sequences (31), pairing double nicking (32) and modifying Cas9 (33–35). All of these efforts were aimed at decreasing the binding affinity between Cas9/sgRNA and off-target sites. However, as long as Cas9 and guide RNA are both present in the cells, they will keep editing off-target sites. Because genome editing is irreversible, the indels at off-target sites will accumulate at the population level, especially after cells are passaged for many generations *in vitro* or *in vivo*. On the other hand, removal of Cas9 after achieving maximal on-target editing will avoid the accumulation of off-target indels, which will lead to diminished off-target effects. A DOX-iCas9 system allows for temporal control of Cas9 and shorter exposure of DNA at off-target sites to the endonuclease. Our data showed that shortening the exposure of the genome to Cas9/sgRNA significantly decreased off-target effects (Figure 4). This is in concordance with observations made by researchers by applying different Cas9 inducible systems (13,15) or controlling the amount of Cas9 protein (51). However, the gene silencing efficiencies with those systems are not as high as the DOX-controlled system we are reporting. The sensitivity and ease of our system is balanced with high gene silencing efficiency and low off-target effects. More importantly, our strategy can be combined with other existing strategies, such as truncated guide sequences or engineered Cas9, to further minimize potential off-target effects.

## Supplementary Material

SUPPLEMENTARY DATA
