# Supplementary material for: An easy and efficient inducible CRISPR/Cas9 platform with improved specificity for multiple gene targeting
Source: Nucleic Acids Res. 2016 Jul 25;44(19):e149. doi: 10.1093/nar/gkw660 (PMC5100567; doi:10.1093/nar/gkw660)
Supplement: SUPPLEMENTARY DATA [file supp_44_19_e149__index.html]

An easy and efficient inducible CRISPR/Cas9 platform with improved specificity for multiple gene targeting — SUPPLEMENTARY DATA 

# An easy and efficient inducible CRISPR/Cas9 platform with improved specificity for multiple gene targeting

## SUPPLEMENTARY DATA

- SUPPLEMENTARY DATA
