# Supplementary material for: An easy and efficient inducible CRISPR/Cas9 platform with improved specificity for multiple gene targeting
Source: Nucleic Acids Res. 2016 Jul 25;44(19):e149. doi: 10.1093/nar/gkw660 (PMC5100567; doi:10.1093/nar/gkw660)
Supplement: SUPPLEMENTARY DATA [file supp_gkw660_nar-01425-met-h-2016-File006.pdf]

Supplementary data

for

An easy and efficient inducible CRISPR/Cas9 platform with improved specificity for multiple gene targeting

Jian Cao[1], Lizhen Wu[1], Shang-Min Zhang[1], Min Lu[1,2], William K.C. Cheung[1], Wesley Cai[1], Molly Gale[1], Qi Xu[1,3], and Qin Yan[1*]

[1]Department of Pathology, Yale School of Medicine, New Haven, CT.

[2]Harbin Institute of Technology, Harbin, China

[3]Department of Oncology, Renji Hospital, Shanghai Jiao Tong University School of Medicine, Shanghai, China

*To whom correspondence should be addressed: Tel: +1-203-785-6672; Fax: +1-203-785-2443; Email: qin.yan@yale.edu

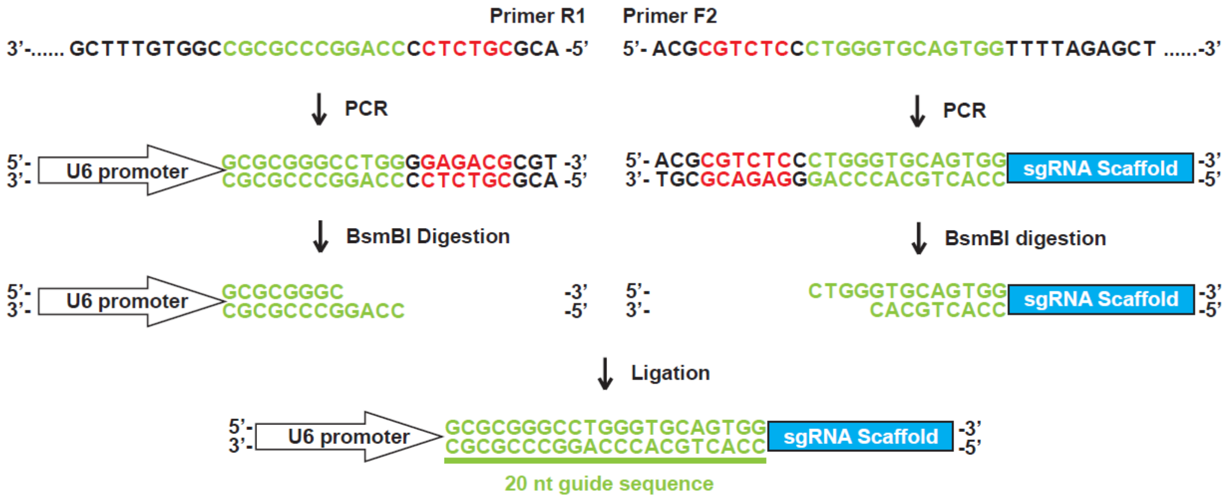

Figure S1. An example of assembly of guide sequence from two primers.

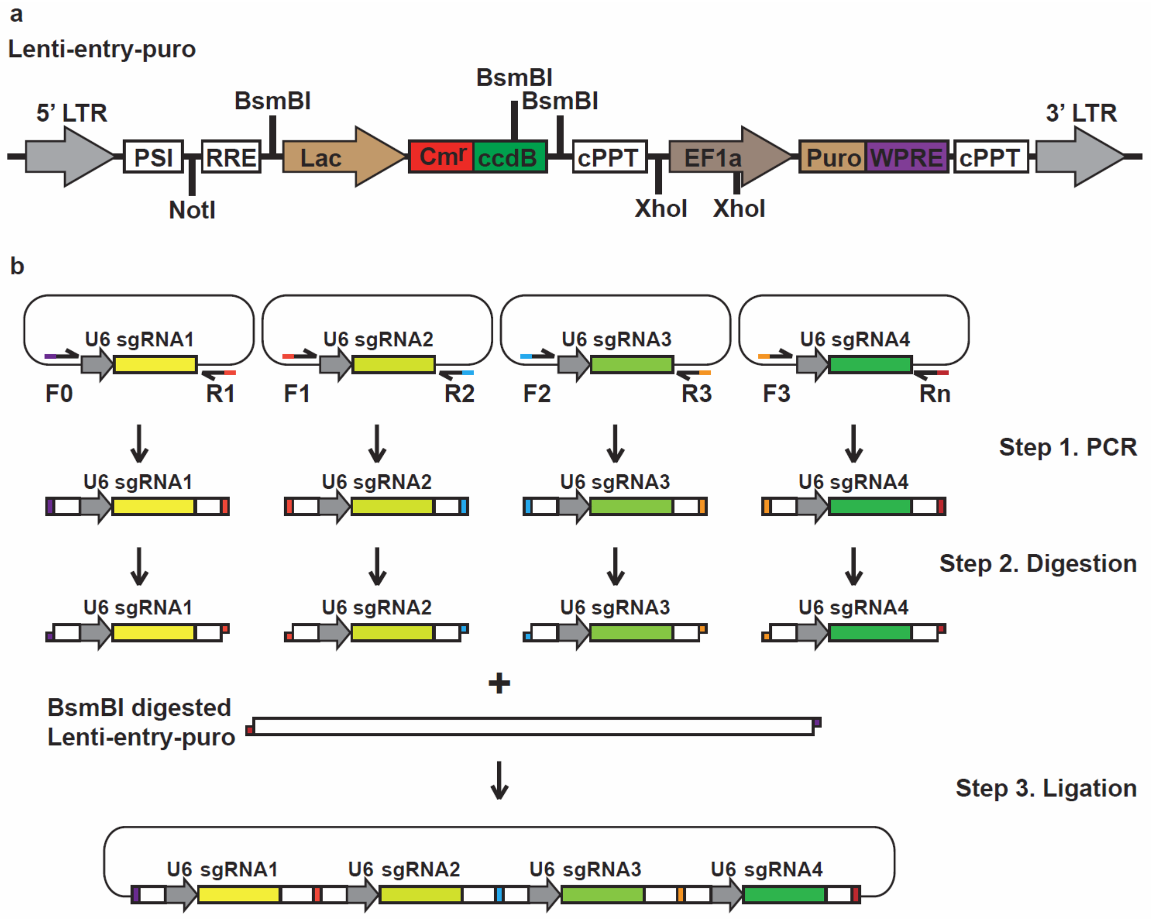

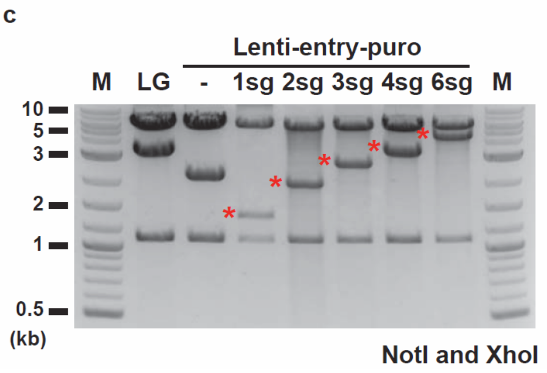

d

Construct	No. of clones	Verified correct clones
1 sgRNA	628	10/10
2 sgRNA	280	10/10
3 sgRNA	179	9/10
4 sgRNA	84	6/10
5 sgRNA	69	4/10
6 sgRNA	54	2/10

Figure S2. A one-step method to generate a multiple sgRNA delivery plasmid using sgRNA delivery plasmid as the template. (a) Schematic representation of the Lenti-entry-puro plasmid. LTR, long terminal repeat; PSI, retroviral Ψ packaging element; RRE, Rev response element; Lac, Lac promoter; Cm^r, Chloramphenicol resistance; ccdB, toxin ccdB gene; cPPT, central polypurine tract; EF1α, EF1α promoter; Puro, puromycin resistance; WPRE, Woodchuck hepatitis virus posttranscriptional regulatory element. (b) Overview of the cloning strategy. (c) NotI and XhoI digestion of indicated plasmids showing correct vector assembly of 1, 2, 3, 4, or 6 sgRNA cassettes. LG, LentiGuide. M, Log-2 DNA ladder (NEB). Fragments carrying sgRNA cassettes are indicated by red stars (*). (d) A summary of the cloning efficiency for making the constructs with different numbers of sgRNAs. 10 clones were randomly picked and verified by the size of NotI and XhoI digested fragments.

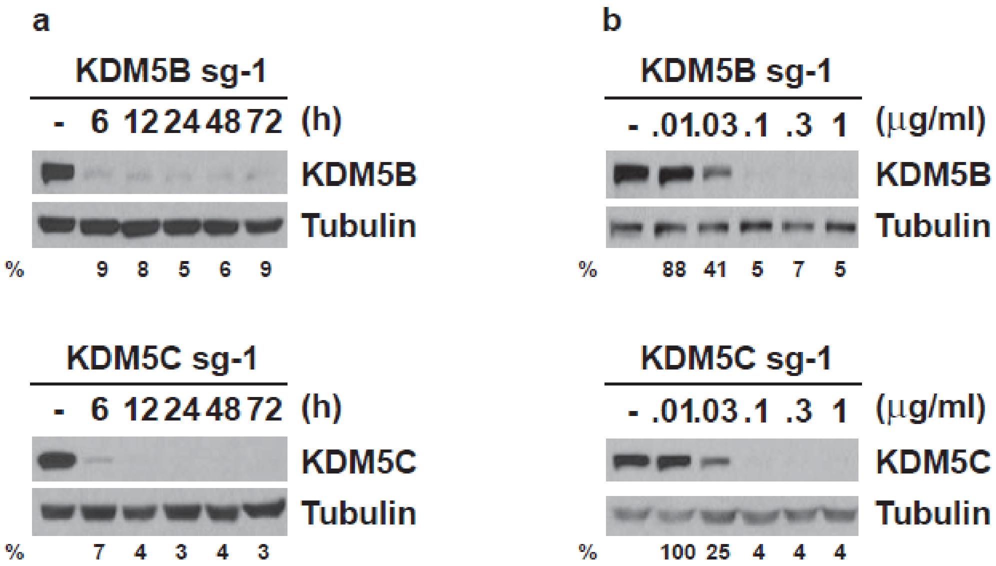

Figure S3. Optimization of induction conditions.

(a) HeLa/iCas9-c1 cells were transduced with KDM5B sg-1 or KDM5C sg-1, treated with 1 µg/ml doxycycline for the indicated time, after which doxycycline was withdrawn, and all cells were harvested after 72 hours for western blot analyses. (e) HeLa/iCas9-c1 were cells transduced with KDM5B sg-1 or KDM5C sg-1, treated with the indicated concentration of doxycycline for 48 hours, and harvested for western blot analyses.

Table S1. Stable inducible Cas9 cell lines.

iCas9 cell lines	Parental cell lines	Unsorted	Sorted
HeLa/iCas9	Human cervical adenocarcinoma cells	+	+
MCF7/iCas9	Human breast adenocarcinoma cells	+	+
SKBR3/iCas9	Human breast adenocarcinoma cells	+	+
BT474/iCas9	Human breast ductal carcinoma cells	+	+
MCF10A/iCas9	Human immortalized breast epithelial cells	+	+
PC9/iCas9	Human lung adenocarcinoma cells	+	
NT2/iCas9	Mouse breast tumor cells	+	

Table S2. Primers used for multiple sgRNA assembly (for Figure 2). BsmBI recognition sites are highlighted in green. sgRNA sequences (F1 and Rn) and splitted sgRNA sequences (R1 and F2, R2 and F3, R3 and F4, R4 and F5) are highlighted in blue. Overhang sequences are underlined. Overhang sequences for inserting into vectors are highlighted in red.

F1	ACG CGTCTC ACACCGCTGCAAAATTCGGCCGCCCAGTTTTAGAGCTAGAAATAGCAAGTT
R1	ACG CGTCTC CCCAGGCCCGCGCCGGTGTTTCGTCCTTTCCAC
F2	ACG CGTCTC CCTGGGTGCAGTGGTTTTAGAGCTAGAAATAGC
R2	ACG CGTCTC GAGCCGGGGTCCGACCGGTGTTTCGTCCTTTCCAC
F3	ACG CGTCTC CGGCTCCATGGGTTTTAGAGCTAGAAATAGC
R3	ACG CGTCTC TTCACCGGAAATCCGGTGTTTCGTCCTTTCCAC
F4	ACG CGTCTC GGTGAAGGATGGGGTTTTAGAGCTAGAAATAGC
R4	ACG CGTCTC GCAGACTGGCATCCGGTGTTTCGTCCTTTCCAC
F5	ACG CGTCTC GTCTGCTCGGCTAGTTTTAGAGCTAGAAATAGC
Rn	ACG CGTCTC AAAACCCGTTCTACATTGGGAATCTCGGTGTTTCGTCCTTTCCAC

Table S3. Primers used for multiple sgRNA assembly (for Figure S2). BsmBI recognition sites are highlighted in green. Overhang sequences are underlined. Overhang sequences for inserting into vectors are highlighted in red.

F1	ACG CGTCTC AGATA GGCAAGTTTGTGGAATTGGT
R1	ACG CGTCTC AGGAC TCTTTCCCCTGCACTGTACC
F2	ACG CGTCTC AGTCC GGCAAGTTTGTGGAATTGGT
R2	ACG CGTCTC ACGAA TCTTTCCCCTGCACTGTACC
F3	ACG CGTCTC ATTCG GGCAAGTTTGTGGAATTGGT
R3	ACG CGTCTC AGCAT TCTTTCCCCTGCACTGTACC
F4	ACG CGTCTC AATGC GGCAAGTTTGTGGAATTGGT
R4	ACG CGTCTC AAGCA TCTTTCCCCTGCACTGTACC
F5	ACG CGTCTC ATGCT GGCAAGTTTGTGGAATTGGT
R5	ACG CGTCTC ATCCG TCTTTCCCCTGCACTGTACC
F6	ACG CGTCTC ACGGA GGCAAGTTTGTGGAATTGGT
R6	ACG CGTCTC ATTGT TCTTTCCCCTGCACTGTACC

Table S4. sgRNAs sequences used in the paper. Point mutations are labeled in red.

Control sg-1	GACCGGAACGATCTCGCGTA
KDM5A sg-1	CGTCTTTGAGCCGAGTTGGG
KDM5B sg-1	GATGCCAGTCTGCTCGGCTA
KDM5B sg-1 mutant	GATGCCAGTATGCTCGGCTA
KDM5C sg-1	GGTTTACCCCCCGAATCCAG
KDM5C sg-1 mutant	GGTTTACCCACCGAATCCAG
KDM5C sg-2	GTCGGACCCCGGCTCCATGG
KDM5C sg-2 mutant	GTCGGACCCAGGCTCCATGG
KDM5C sg-3	AGATTCCCAATGTAGAACGG
KDM5C sg-3 mutant	AGATTCCCACTGTAGAACGG
ARID2 sg-1	CTAATTTACTGCAAGTCGAG
mKDM5B sg-1	AGACTGGGATCTGTAAGGTG
EMX1 sg-1	GAGTCCGAGCAGAAGAAGAA
VEGFA sg-1	GACCCCCTCCACCCCGCCTC

Table S5. Primer sequences for T7 endonuclease assays.

EMX1-on-target-F	CCATCCCCTTCTGTGAATGT
EMX1-on-target-R	GGAGATTGGAGACACGGAGA
EMX1-off-target-F	CCTCCTGAGTTTCTCATCTGTGC
EMX1-off-target-R	CATTGCTTGTCCCTCTGTCAATG
VEGFA-on-target-F	TGAGTGACCTGCTTTTGGGG
VEGFA-on-target-R	GTTCATGGTTTCGGAGGCCC
VEGFA-off-target-F	TACCCACCTCCCTATCCTCAAA
VEGFA-off-target-R	CCTCGCTATTCAAGTGTTGGTG
